# A systematic review of the use of the Consolidated Framework for Implementation Research

**DOI:** 10.1186/s13012-016-0437-z

**Published:** 2016-05-17

**Authors:** M. Alexis Kirk, Caitlin Kelley, Nicholas Yankey, Sarah A. Birken, Brenton Abadie, Laura Damschroder

**Affiliations:** 1 Department of Health Policy and Management, Gillings School of Global Public Health, The University of North Carolina at Chapel Hill, Chapel Hill, NC 27599-7411 USA; 2 Performance Measure Development and Implementation Program, RTI International, 3040 Cornwallis Road, Research Triangle Park, NC 27709 USA; 3 VA Health Services Research & Development Center for Clinical Management Research, VA Ann Arbor Healthcare System, P.O. Box 130170, Ann Arbor, MI 48113-0170 USA; 4 Psychology Department, Eastern Michigan University, 341 Science Complex, Ypsilanti, MI 48197 USA

**Keywords:** Implementation research, Theory, Frameworks, Synthesis, Consolidated Framework for Implementation Research

## Abstract

**Background:**

In 2009, Damschroder et al. developed the Consolidated Framework for Implementation Research (CFIR), which provides a comprehensive listing of constructs thought to influence implementation. This systematic review assesses the extent to which the CFIR’s use in implementation research fulfills goals set forth by Damschroder et al. in terms of breadth of use, depth of application, and contribution to implementation research.

**Methods:**

We searched Scopus and Web of Science for publications that cited the original CFIR publication by Damschroder et al. (Implement Sci 4:50, 2009) and downloaded each unique result for review. After applying exclusion criteria, the final articles were empirical studies published in peer-review journals that used the CFIR in a meaningful way (i.e., used the CFIR to guide data collection, measurement, coding, analysis, and/or reporting). A framework analysis approach was used to guide abstraction and synthesis of the included articles.

**Results:**

Twenty-six of 429 unique articles (6 %) met inclusion criteria. We found great breadth in CFIR application; the CFIR was applied across a wide variety of study objectives, settings, and units of analysis. There was also variation in the method of included studies (mixed methods (*n* = 13); qualitative (*n* = 10); quantitative (*n* = 3)). Depth of CFIR application revealed some areas for improvement. Few studies (*n* = 3) reported justification for selection of CFIR constructs used; the majority of studies (*n* = 14) used the CFIR to guide data analysis only; and few studies investigated any outcomes (*n* = 11). Finally, reflections on the contribution of the CFIR to implementation research were scarce.

**Conclusions:**

Our results indicate that the CFIR has been used across a wide range of studies, though more in-depth use of the CFIR may help advance implementation science. To harness its potential, researchers should consider how to most meaningfully use the CFIR. Specific recommendations for applying the CFIR include explicitly justifying selection of CFIR constructs; integrating the CFIR throughout the research process (in study design, data collection, and analysis); and appropriately using the CFIR given the phase of implementation of the research (e.g., if the research is post-implementation, using the CFIR to link determinants of implementation to outcomes).

**Electronic supplementary material:**

The online version of this article (doi:10.1186/s13012-016-0437-z) contains supplementary material, which is available to authorized users.

## Background

A top priority for implementation research is to understand why an innovation is successfully implemented in one setting, but not in another. Without a theoretical framework to guide data collection, analysis, and interpretation, implementation researchers often identify determinants of implementation that apply only to the specific contexts in which their research was conducted. Conducting implementation research without a theoretical framework also hinders a foundational scientific goal of being able to generalize and build on findings across studies and contexts. Consequently, researchers and policymakers have called for greater use of theory in implementation research [1]. Many implementation theoretical frameworks describe similar or overlapping constructs, each with slightly different terminologies and definitions [2]. Thus, in 2009, Damschroder et al. undertook a review of the implementation science literature with the aim of integrating previously published theories into a single, consolidated framework to guide implementation research [3]. The result of this literature review was the Consolidated Framework for Implementation Research (CFIR).

The CFIR is a meta-theoretical framework that provides a repository of standardized implementation-related constructs that can be applied across the spectrum of implementation research [3]. The CFIR comprises 39 constructs organized across five major domains, all of which interact to influence implementation and implementation effectiveness [3]. Additional file 1 includes a description of CFIR constructs within each domain.

The CFIR provides a common language by which determinants of implementation can be articulated, as well as a comprehensive, standardized list of constructs to serve as a guide for researchers as they identify variables that are most salient to implementation of a particular innovation [4]. The CFIR can be used to develop data collection approaches (e.g., interview guide, codebook) and as a guide for analyzing, interpreting, and/or reporting implementation-related findings. The CFIR can be applied at any phases of implementation (i.e., pre-, during, or post-implementation), and researchers can also use the CFIR’s constructs as building blocks for developing testable hypothetical models that focus on specific constructs and their interrelationships. At the macro level, the CFIR provides a standardized structure for building on findings across studies [3].

Although use of the CFIR as a theoretical framework appears to be on the rise since its publication in 2009, no formal reviews have been conducted to investigate its impact on implementation research. Thus, our specific research objectives for this systematic review are as follows:

- Objective 1: determine types of studies that use the CFIR.
- Objective 2: determine how the CFIR has been applied, including depth of application.
- Objective 3: determine the contribution of the CFIR to implementation research.

The objectives of this systematic review are based on a review of Damschroder et al.’s seminal publication [3], which specified criteria for using the CFIR and goals for the CFIR over time. Findings from this systematic review can be used as a reference for implementation researchers as they integrate the CFIR into their work. To this end, we conclude this systematic review by making recommendations to promote the CFIR’s use as intended by Damschroder et al.

## Methods

### Search strategy

We employed a citation search strategy to identify published peer-reviewed articles that describe use of the CFIR to guide their research. The cited article used for our search was the original 2009 CFIR publication by Damschroder et al. [3]. We searched two citation index databases, Web of Science and Scopus, from August 2009 through January 2015. These databases were selected because they offer the most comprehensive databases of articles that can be tracked using citations, and they allow for cited reference searching. Although other databases, such as Google Scholar, may provide wider coverage of certain types of publications (international, non-English journals, conference proceedings) [5], those publications were excluded from our review. In addition, literature shows that Web of Science and Scopus yield more consistent and accurate results than other databases that may provide wider coverage (e.g., Google Scholar) [6]. In Scopus, the search string was REF (fostering implementation of health services research findings into practice: a consolidated framework for advancing implementation science). In Web of Science, the search strategy was TITLE: (fostering implementation of health services research findings into practice: a consolidated framework for advancing implementation science), and the “times cited” link provided a full list of citations of the original CFIR paper. Full texts of all resulting articles were downloaded for review.

### Inclusion and exclusion criteria

English language research that used the CFIR in a meaningful way (i.e., use of CFIR to guide data collection, measurement, coding, analysis, and/or reporting) in an empirical study and were published in a peer-reviewed journal were included. Excluded were protocols, editorials, and articles that cited the CFIR, but neither reported applying nor planning to apply the CFIR (e.g., cited the CFIR in the introduction to acknowledge the complexity of implementation context). This review focused on empirical studies, so we excluded syntheses in which the CFIR was one of a list of frameworks, theories, or models.

### Study selection process

Article selection was performed by two authors (CK, AK). One author (CK) performed a full text analysis of all articles, searching for evidence of meaningful use of the CFIR by examining the methods and results sections. There was no initial screening based on titles or abstracts because this review is based on citations of the CFIR, and the CFIR was not always mentioned in those sections. Before the primary reviewer (CK) conducted the full text review of all articles, the research team went through a preliminary review process to refine inclusion and exclusion criteria. During this preliminary review process, a second author (AK) reviewed of a sub-sample of articles (71 articles, or 17 % of 429 unique papers resulting from our search) to establish refined inclusion and exclusion criteria, and ensure criteria were applied consistently. During this preliminary phase, if the reviewers disagreed or were uncertain about the inclusion of any article, the article was discussed with the senior authors (LD, SB). After the preliminary review phase, CK completed a full-text review of all articles. During this phase, the majority of articles were excluded because they did not meet the criteria for using the CFIR in a meaningful way. All articles marked for inclusion were also assessed by a second reviewer (AK). The two reviewers achieved 80 % agreement on their determination of whether to include articles in the final sample. When there was disagreement, both reviewers discussed until consensus was reached.

### Data extraction and analysis

We used a framework analysis approach to guide abstraction and synthesis of the included articles [7]. The study team developed a standardized data abstraction tool in Microsoft Excel where content was arrayed in a matrix format consisting of rows (articles), columns (codes), and cells (summarized data) [8]. Abstraction was accomplished in five phases. First, in the familiarization phase, we reviewed a subset of the included articles to familiarize team members with the literature base. Second, we identified a thematic framework based on our specific research objectives, which served as the codes used to identify and extract passages from the articles. These codes comprised the columns in our abstraction matrix. Final codes by study objective are presented in Table 1.

**Table 1:** Research objectives and operationalization

Research objective	Operationalization of objective (codes for analysis)
Objective 1: determine the types of studies that use the CFIR	General study characteristics, including: • Research objective • Setting • Unit of analysis (e.g., organization- or provider-level) • Phase of implementation (pre-, during, or post-implementation) • Study design and methods (qualitative, quantitative, or mixed-methods)
Objective 2: determine how the CFIR is being applied, including depth of application	Depth of CFIR application, including: • How the CFIR was used (e.g., to guide data analysis, data collection, or both) • Rationale of selection of CFIR constructs, as well as which CFIR constructs were selected and used • Investigation of outcomes, including implementation effectiveness outcomes, and measurement of association between CFIR constructs and outcomes
Objective 3: determine the contribution of the CFIR to implementation research	General commentary about validity and utility of the CFIR, based on three questions posed by Damschroder et al. in their seminal CFIR publication [3], which included: • Coherence of CFIR terminology • Whether the CFIR promotes comparison across studies • Whether the CFIR stimulates new theoretical development

In the third and fourth phases, indexing and charting, four authors (CK, AK, NY, and BA) extracted text selections from included articles into our abstraction matrix. Two individuals independently indexed and charted each article, comparing results; discrepancies were discussed until consensus was reached. In the fifth and final phase, mapping and interpretation, passages from the abstraction matrix were analyzed by AK, SB, and LD to develop overarching themes for each code. Themes were discussed among all co-authors until consensus was reached.

The quality and depth of application of the CFIR was assessed by (1) the inclusion criteria of our systematic review (i.e., the proportion of articles that were included because they used the CFIR in a meaningful way) and (2) research objective 2, which focuses on the depth of CFIR application. This assessment was intended to evaluate quality of CFIR application and not the quality of any other aspects of the studies described in the included articles.

## Results

**Fig. 1:**
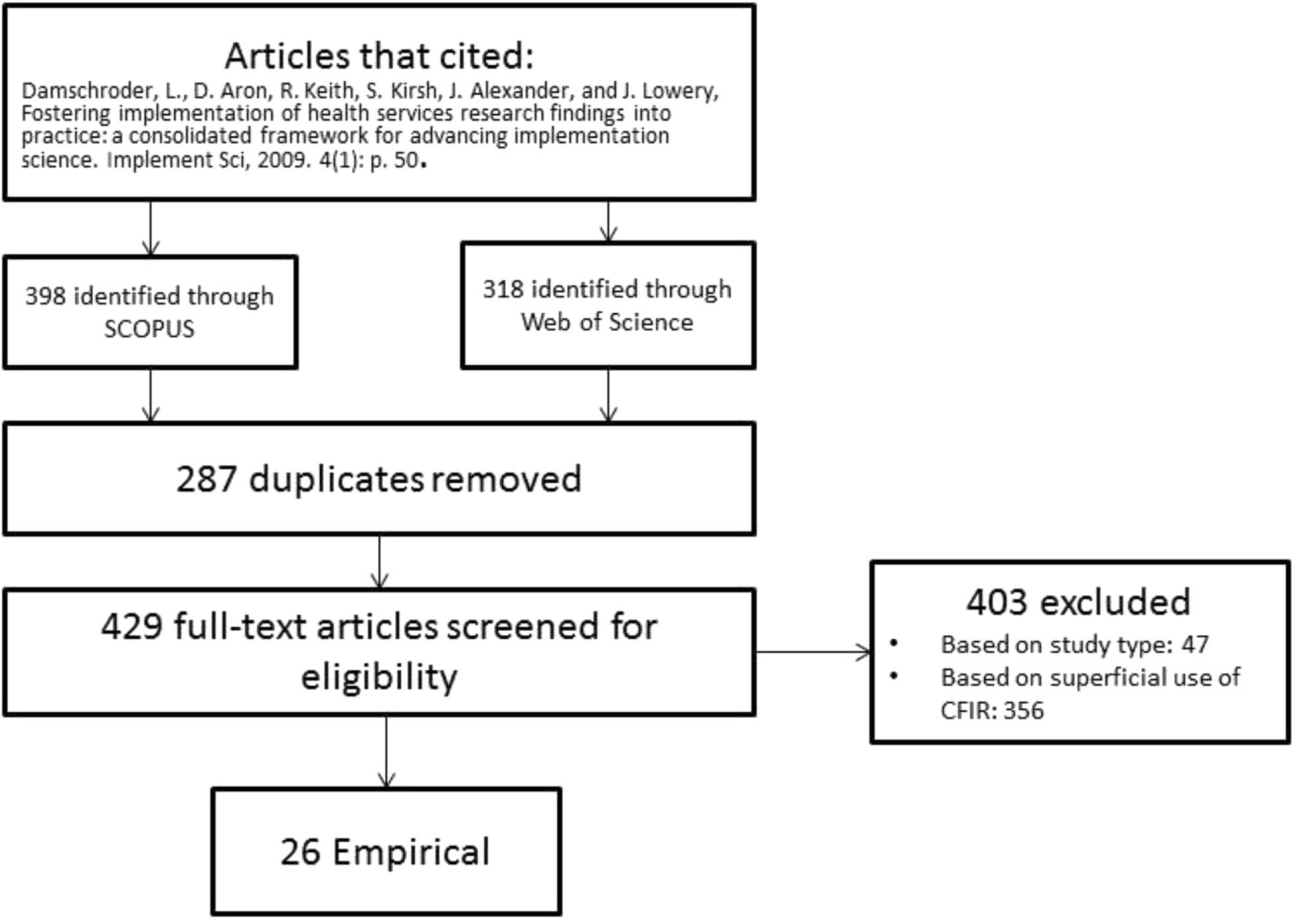
PRISMA diagram of study selection

Our searches yielded 716 retrievals; the search in Scopus yielded 398 retrievals and Web of Science yielded 318. Of those 716, 287 were duplicate retrievals and were removed, leaving 429 unique articles that cited the 2009 CFIR paper. Of the 429 unique articles, we excluded a total of 403 articles. Three hundred fifty-six articles were excluded because they were non-meaningful uses of the CFIR (e.g., use of the CFIR as background or plans for future research in the discussion), and 47 were excluded because they were syntheses, study protocols, etc.; 26 articles (6 % of 429) were included in the final sample. See Fig. 1. All 26 included articles were reviewed to meet our three research objectives.

### Objective 1: types of studies that have used the CFIR

Each study was characterized with respect to research objective, setting, unit of analysis, phase of implementation, and study design and methods (see Table 2).

**Table 2:** Summary of included studies

Author	Research objective	Methods	Unit of analysis	Phase of implementation
Acosta et al. (2013) [33]	Assess impact of Assets-Getting to Outcomes intervention on individual prevention practitioners and whole prevention programs in 12 Maine communities	Mixed	Program (program and coalition)	Post
Baker et al. (2014) [34]	Investigate mental health care workers’ views of a physical health self-management program in South Australia	Qualitative	Provider	Post
Balas et al. (2013) [35]	Implementation of the awakening and breathing coordination, delirium monitoring/management, and early exercise/mobility bundle in a tertiary care setting	Mixed	Provider (clinician)	Post
Cilenti et al. (2012) [11]	Identify factors of successful implementation of evidence-based practices in public health agencies	Qualitative	Department	Post
Connell et al. (2014a) [18]	Survey therapists’ use of Graded Repetitive Arm Supplementary Program for upper limb stroke rehabilitation in Vancouver, British Columbia	Quantitative	Provider (therapists)	Post
Connell et al. (2014b) [36]	Implementation of Graded Repetitive Arm Supplementary Program for upper limb stroke rehabilitation in Vancouver, British Columbia	Qualitative	Provider	Post
Cragun et al. (2014) [19]	Explore how variation in universal tumor screening procedures for colorectal cancer patients influenced patient follow through with germ-line testing after a positive result	Mixed	Organization	Post
Damschroder and Lowery (2013) [20]	Identify factors affecting implementation of the MOVE! weight management program in Veterans Affairs medical centers	Mixed	Provider	Post
Draanen et al. (2013) [37]	Examine effectiveness of Toronto Community Addictions Team on service and substance use in Toronto	Mixed	Provider (individual)	During
English et al. (2011) [38]	Implementation of a multifaceted intervention to improve inpatient care in rural Kenyan hospitals	Mixed	Organization (hospital)	Post
English et al. (2013) [22]	Design of a tailored intervention strategy to improve hospital services for children in Kenya	Qualitative	NA (designing intervention for hospitals)	Pre
Forman et al. (2014) [17]	Understanding experiences of primary care leadership, physicians, and staff during Patient Aligned Care Teams early implementation in Veterans Affairs medical centers	Qualitative	Provider (individual provider/staff)	During
Gilmer et al. (2013) [39]	Implementation of full service partnerships, supportive housing programs for persons with serious mental illness in California	Mixed	Program	During
Green et al. (2014) [23]	Examine the adoption and use of buprenorphine for opioid addiction treatment in two not-for-profit health plans	Qualitative	Provider	During
Ilott et al. (2012) [12]	Testing the CFIR through post hoc analysis of 11 narrative accounts of health care service and practice innovation in England	Qualitative	Organization	Post
Jones et al. (2015) [40]	Implementation of central line associated bloodstream infections reduction project in an orthopedic and trauma surgical unit in an academic health care system in the southeast region of the United States	Quantitative	Organization (hospital unit)	Post
Kalkan et al. (2014) [9]	Explore the influences on individual rheumatologist’s decisions on prescribing biological drugs in Sweden	Mixed	Provider	Not specified
Kilbourne et al. (2013) [21]	Measure success of randomized adaptive implementation trial to improve uptake of a re-engagement program for patients with serious mental illness in Veterans Affairs medical centers	Mixed	Organization and patient	During
Luck et al. (2014) [16]	Evaluation of Patient Aligned Care Teams toolkit across Veterans Affairs medical centers	Mixed	Organization	Post
Robins et al. (2013) [10]	Investigated potential facilitators and barriers to applying a blood pressure management strategy in a community setting.	Qualitative	Providers and patient	Pre
Ruffolo and Capobianco (2012) [13]	Investigate the implementation of a family psychoeducation intervention into routine care throughout an entire state.	Qualitative	Organization	Post
Sanchez et al. (2014) [41]	Examine medication reconciliation implementation in a large academic medical center and its affiliated Veterans Affairs medical center.	Qualitative	Organization	Post
Shaw et al. (2013) [42]	Examine engagement of health care providers in the implementation of a fall prevention program.	Mixed	Provider	During
Shimada et al. (2013) [14]	Explore variations in adoption and outcomes of patient-provider secure messaging in Veterans Affairs medical centers.	Quantitative	Organization	During
Zulman et al. (2013) [43]	Evaluate the large-scale implementation of an internet mediated walking program delivered by a large US health insurance company.	Mixed	Organization	Post
Zulman et al. (2014) [15]	Evaluate a healthcare delivery redesign process for high-need, high-cost patients in Veterans Affairs medical centers.	Mixed	Organization	During
Total *n* = 26	NA	Qualitative: 10 Quantitative : 3 Mixed: 13	Organization: 10 Provider: 10 Program: 2 Department: 1 Provider and patient: 1 Organization and patient: 1 NA: 1	Pre: 2 Post: 15 During: 8 Not specified: 1

#### Research objective

All but one study [9] investigated facets of implementation of innovations that had already been developed and tested for feasibility or effectiveness. The general research objective of most (73.1 %) studies was to gain an in-depth understanding of practitioners’ experiences (e.g., implementation processes, barriers and facilitators to implementation) in innovation implementation. Studies investigated implementation of a wide variety of innovations (e.g., healthcare delivery and process re-design, quality improvement, health promotion, and disease management), spanning a wide variety of health-related topics (e.g., mental health, obesity, and blood pressure).

#### Setting and unit of analysis

Healthcare systems were the most common settings for implementation (*n* = 20). The unit of analysis was most often the organization in which implementation occurred (*n* = 12) or the providers involved in implementation (*n* = 11); other units of analysis included programs (*n* = 2), departments (*n* = 2), and patient (*n* = 2).

#### Phase of implementation

The majority of studies (*n* = 15) focused their data collection and evaluations in the post-implementation phase, though some studies did occur during (*n* = 8) or pre-implementation (*n* = 2). In the seminal CFIR publication, Damschroder et al. outlined ways in which the CFIR could be used across phases of implementation [3]. For pre-implementation research, the CFIR provides a list of “explicitly defined constructs” to conduct capacity and needs assessments to identify potential determinants (barriers and facilitators) to implementation [3]. During implementation, CFIR constructs can provide a roadmap to monitor implementation progress. In post-implementation, the CFIR can be used to “guide exploration” to determine what factors influenced outcomes such as implementation effectiveness and intervention effectiveness [3]. In general, we found that phase of implementation mapped onto Damschroder et al.’s intended use of the CFIR, though there were some exceptions, especially among post-implementation studies. For example, studies that occurred during the pre-implementation phase used the CFIR to investigate potential barriers to implementation prior to the roll-out of the innovation. A case in point, Robins et al. used the CFIR to guide interviews of providers about potential barriers to implementing a home blood pressure monitoring program in the pre-implementation phase [10]. Findings revealed that there was a flaw in a key component of the program; specifically, many clinics lacked an in-house pharmacist necessary for the program (coded to Available Resources within the Inner Setting). Subsequently, the investigators explored options for re-designing the program to accommodate clinics without an in-house pharmacist.

Some studies, however, did not apply the CFIR in a manner completely consistent with the guidance set forth by Damschroder et al. [3]. In particular, studies that occurred post-implementation most commonly used the CFIR to investigate facilitators/barriers to implementation among participants who had already adopted and implemented an innovation (e.g., [11]), thus identifying determinants of implementation post hoc. This application of the CFIR is more aligned with Damschroder et al.’s pre-implementation guidance and does not incorporate a key component of Damschroder et al.’s suggestion for applying the CFIR to post-implementation research—which is to link determinants of implementation to outcomes (e.g., implementation or innovation effectiveness).

#### Study design and methods

The majority of studies employed either a mixed methods (*n* = 13) or qualitative (*n* = 10) design. Only three studies were purely quantitative. Common qualitative methods included key informant interviews and focus groups with program managers or participants. Studies using quantitative methods commonly employed surveys and administrative data collection methods. Quantitative measures of CFIR constructs were developed using a variety of outside sources because no widely accepted quantitative measures for CFIR constructs currently exist. Most studies engaged in primary data collection either through interviews or surveys. Two studies, however, conducted secondary analysis of existing qualitative data sources including meeting minutes, memos, and quarterly reports generated during the implementation of the innovation under investigation [12, 13].

### Objective 2: depth of CFIR application

As an initial overall indicator of the depth of CFIR application, it is interesting to note that of the initial 429 unique articles that cited the original CFIR publication, 356 (82.9 %) did not meet our definition for meaningful use of the CFIR (i.e., they did not integrate the CFIR in methods and reporting of findings). Among the 26 articles that did meet our inclusion criteria, we assessed the depth of CFIR application according to the following criteria: selection and use of CFIR domains and constructs, application of the CFIR in the methods, and whether the CFIR was being used to investigate outcomes.

### Selection and use of CFIR domains and constructs

**Fig. 2:**
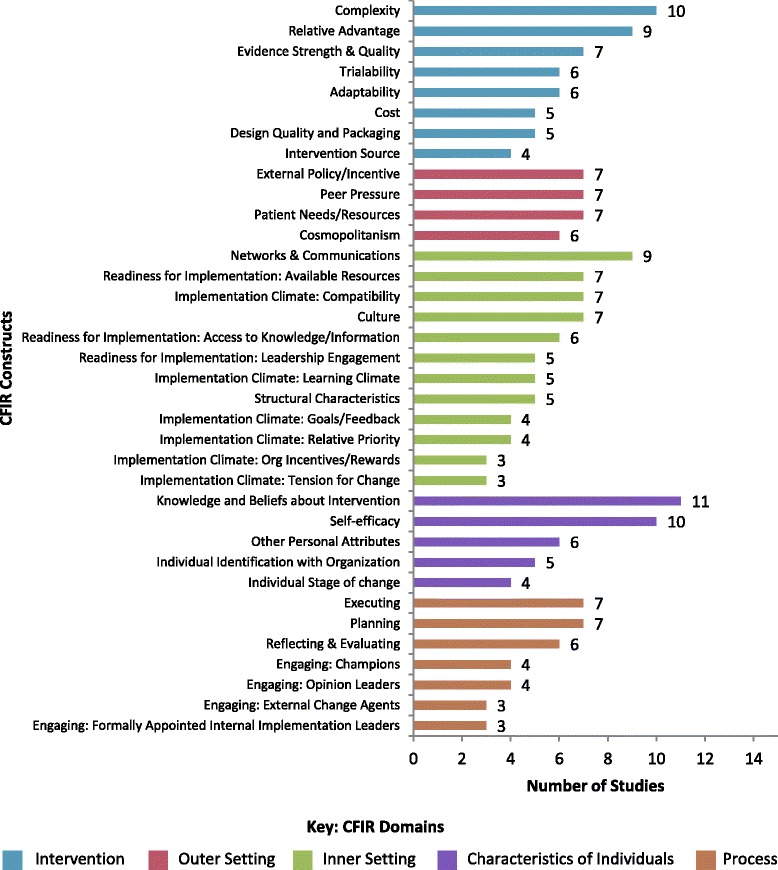
Count of CFIR constructs used in studies. Note: This figure includes only studies which specified CFIR constructs used (*n*=15). Nine studies specified only domains that were used and no constructs. Two studies made no explicit reference to any CFIR domains or constructs

Figure 2 reports CFIR constructs used in studies, rank ordered from highest to lowest use within each domain. (For a listing of which constructs where used by which studies, see Additional file 2.) Overall, there was wide variation in use of CFIR domains and constructs. Some of the included studies reported on domains only (*n* = 9), while others used all domains and all constructs (*n* = 3). Two studies [14, 15] made no explicit reference to use of specific domains or constructs.

With respect to the selection of CFIR constructs, Damschroder et al. offered guidance on how to best select, operationalize, measure, and report findings of CFIR constructs. Damschroder et al. recommended that implementation researchers assess each CFIR construct for salience; determine levels at which each construct should be defined and measured (e.g., individual, team, clinic); and be aware of time points at which each construct is measured [3]. They also recommended that researchers report each decision and rationale, along with findings for each construct that is ultimately selected [3]. In our review, we found that authors of only three articles reported justification for their selection of the set of CFIR domains/constructs used [9, 16, 17]. Within these three articles, there were two main strategies used to select relevant CFIR constructs. Two of the three articles selected CFIR constructs based on their own prior knowledge about which CFIR domains/constructs would be most relevant to their research question [16, 17]. The remaining article [9] selected constructs by first piloting an interview guide that included questions for all CFIR domains. Based on pilot interviews, the authors identified which constructs were deemed most relevant by providers involved in implementation and included only those constructs in the final version of the interview guide.

#### Application of the CFIR in the methods

Table 3 reports application of the CFIR (i.e., whether the CFIR was used to guide data collection, analysis, or both) by study design (mixed methods, quantitative, or qualitative). The majority of included studies (*n* = 14 (54 %)) used the CFIR to guide data analysis only. Three studies used the CFIR to guide data collection only, and six studies used the CFIR to guide both data collection and analysis.

**Table 3:** Application of the CFIR by study design

Study design	Application of the CFIR in methods
Qualitative: *n* = 10	Data collection: 1 (10 %) Data analysis: 7 (70 %) Both: 0 (0 %) Neither: 2 (20 %)
Quantitative: *n* = 3	Data collection: 1 (33.3 %) Data analysis: 0 (0 %) Both: 1 (33.3 %) Neither: 1 (33.3 %)
Mixed methods: *n* = 13	Data collection: 4 (30.8 %) Data analysis: 5 (38.5 %) Both: 3 (23.1 %) Neither: 1 (7.7 %)

The way the CFIR informed study methods depended on study design. For qualitative studies, the CFIR was used to guide data collection via development of semi-structured interview guides or focus group protocols. For qualitative analysis, the CFIR was used to guide development of qualitative coding templates. In quantitative or mixed-method studies, the CFIR was used in the data collection phase to inform survey question development (e.g., [18, 19]). In quantitative or mixed-method analysis, the CFIR was used in a variety of ways. In mixed-method approaches (e.g., [9]), the CFIR was used with both qualitative and quantitative methods to ascertain complementary information about implementation-related efforts. For example, Kalkan et al. [9] used the CFIR qualitatively to elicit information about CFIR constructs which providers believed had any influence on implementation; quantitative ranking of the most influential CFIR constructs allowed the researchers to ascertain quantitative impact of various CFIR constructs. Quantitative uses of the CFIR mostly focused on linking CFIR constructs to implementation or innovation outcomes. For example, Damschroder and Lowery [20] used quantitative ratings of CFIR constructs to distinguish between facilities with high and low implementation effectiveness, and found that 10 CFIR constructs strongly distinguished between high and low implementation effectiveness.

Authors reported that using the CFIR to guide data collection efforts had advantages over using the CFIR in data analysis only. Authors that used the CFIR to inform the development of their structured interview guides stated that if they had not used the CFIR constructs as a “checklist” of variables for consideration, they would have either missed factors to assess, or they would have asked about irrelevant factors [9, 17]. Conversely, investigators reported that using the CFIR only to guide data analysis was a disadvantage because they did not anticipate the importance of unmeasured implementation factors.

#### Investigation of outcomes using the CFIR

The majority of articles (*n* = 15 (58 %)) did not investigate outcomes and focused primarily on descriptive objectives to identify barriers and facilitators of implementation. Slightly less than half (*n* = 11 (42 %)) of the included articles assessed implementation outcomes, which varied widely. Some studies investigated implementation or innovation effectiveness as the outcome of interest, while other studies investigated process outcomes (e.g., exposure to the innovation). Of the 11 studies that investigated outcomes, six assessed associations between CFIR constructs and relevant outcomes. Application of the CFIR to investigate outcomes included using CFIR constructs to distinguish between high and low implementation effectiveness, using CFIR constructs as predictors of implementation effectiveness outcomes in regression models, and using CFIR constructs as control variables in analysis. Kilbourne at al. [21] is an example of the latter application (using CFIR constructs as controls), and is the most extensive quantitative analytic approach included in our sample (i.e., the only article that developed a predictive analytic model). Kilbourne et al. used quantitative measures of 12 CFIR constructs (e.g., implementation climate, complexity, peer pressure) that were distributed across all five domains as covariates in analytic models to examine changes in implementation outcomes. Quantitative measures of CFIR constructs allowed the authors to control for additional factors beyond typical patient-level characteristics, such as gender, race, and age.

### Objective 3: contribution of the CFIR to implementation research

For Objective 3, our goal was to determine the contribution of the CFIR to implementation research, which we assessed by considering three questions posed by Damschroder et al. in their 2009 article introducing the CFIR [3]. These three questions included (1) coherence of CFIR terminology, (2) whether the CFIR promotes comparison across studies, and (3) whether the CFIR stimulates new theoretical development. Overall, reflections on the CFIR’s utility and contributions within our sample were sparse. The following three sections provide insights based on the articles included in this review.

#### Is CFIR terminology and language coherent?

In general, authors offered few reflections on the coherence of CFIR terminology and constructs. A few authors noted that the constructs were generally easy to understand and apply (e.g., [12, 20–23]); others noted specific suggestions for clarification and some gaps in the constructs. For example, Damschroder and Lowery [20] reported some challenges distinguishing between related constructs in qualitative data coding, but noted that this was overcome by using concrete examples from their own data. They identified these construct pairs, which included relative priority versus patient needs and resources, and design quality and packaging versus access to knowledge and information. To further clarify the distinction between these constructs, Damschroder and Lowery added specific examples to an online CFIR technical assistance interactive wiki (www.cfirguide.org), which they (and other researchers [24]) encourage implementation researchers to use for additional coding guidance and to promote sharing continued refinements to these guides. Ilott and colleagues [12] identified several gaps in the CFIR and recommended more development of a few constructs. Improvements in existing constructs noted by these authors include identification of stakeholders in the tension for change construct, as well as a time point component for relative advantage, since these perceptions may change over time. The primary gap identified by Ilott et al. was lack of consideration of scale-up, spread, and sustainability. Ilott et al. noted that the CFIR process domain ends after reflecting and evaluating, which they noted as “premature given the importance of longer term change” [12].

#### Does the CFIR promote comparison of results across contexts and studies over time?

Few included articles (*n* = 3) compared their findings to other studies. However, the discussion section of several articles noted that the standardized nature of the CFIR would promote comparison across future research. Thus, the potential is acknowledged, although few actually did cross-comparisons.

#### Does the CFIR stimulate new theoretical development?

Only two articles mentioned how the CFIR promoted theoretical development [17, 20]. One article mentioned that further research was needed to develop measures, to propose and test models that predict implementation, and to assess the extent to which constructs can be used to develop implementation strategies [20]; another article linked CFIR constructs to a “foundational strategy for implementation” [17].

## Discussion

Overall, the CFIR has been applied across a wide range of studies with a wide range of objectives, units of analysis, design, and methods, suggesting that the CFIR is applicable to a wide range of interventions, settings, and research designs. Despite the CFIR’s application to a wide range of studies and settings, we found areas for improvement with respect to the depth of the CFIR’s application. These findings are discussed in greater detail below.

### The use and selection of CFIR constructs

Overall, we found wide variation in which CFIR constructs were used and evaluated, and little reporting of methods or logic for selecting CFIR constructs or domains. This gap limits the contribution of the CFIR to implementation science, and is not in alignment with guidance published by Damschroder et al., which recommends that researchers report each decision and rationale for selection, measurement, and reporting of CFIR constructs. A justification of selection of CFIR constructs would help ensure consistency of implementation studies (i.e., that the most salient implementation-related factors were investigated). In addition, we found several instances where authors were not explicit about which constructs were selected (i.e., no reporting of final constructs or reporting was at the domain-level only). This lack of specificity limits opportunities to compare research over time and across contexts.

### How the CFIR was used

The majority of studies (53.8 %) used the CFIR only to guide data analysis. Some authors noted this as a limitation because they did not anticipate the importance of certain unmeasured implementation factors. As the use of the CFIR continues to increase, and as with use of any theory, integrating the CFIR into research question development and data collection efforts early-on will strengthen research and applicability of findings.

### Phase of implementation

Most studies applied the CFIR during- or post-implementation to identify barriers and facilitators to implementation of an innovation. Only two studies (7.69 %) used the CFIR prior to innovation implementation to help inform future implementation efforts. This is a potential missed opportunity since studies that did use the CFIR prior to implementation (e.g., [10]) were able to identify potential barriers to implementation, refine their implementation strategy, and adapt the innovation before implementation began. The use of the CFIR to prospectively investigate implementation issues before a program is rolled out on a large scale can allow for critical program re-design, increasing the likelihood of successful program dissemination and implementation. The use of the CFIR during- or post-implementation may have similar benefits, but only if researchers use information about barriers and facilitators to inform adaptations of the innovation, implementation, scale-up, or sustainment. In general, our review found little evidence of post-implementation findings being applied to help inform implementation, scale-up, or sustainment. Additionally, Damschroder et al. identified that the meaningful use of the CFIR in post-implementation research would include linking CFIR constructs (determinants of implementation) to outcomes (implementation or innovation effectiveness); results revealed that only about half of the post-implementation studies reported investigating the association of CFIR constructs with outcomes. Additional information on investigation of outcomes is reported below.

### Investigation of outcomes using CFIR constructs

Investigation of outcomes is critical for the field of implementation science overall because optimal implementation outcomes are a necessary condition for innovation effectiveness [25, 26]. Additionally, the study and measurement of implementation outcomes is vital since it allows the field to “advance the understanding of implementation processes, enable studies of comparative effectiveness of implementation strategies, and enhance efficiency in implementation research” [25]. Fewer than half of the included studies in our review assessed outcomes and even fewer (*n* = 6) linked CFIR constructs to outcomes. This is a gap in the implementation literature in and of itself and not necessarily constrained to those that reported use of the CFIR. Even among articles that did investigate outcomes, there is room for improvement in outcome metrics since some were just metrics of exposure to an innovation, not meaningful measures of implementation effectiveness, such as those conceptualized by Proctor et al. [25]. In studies that did relate CFIR constructs to outcomes, there was variation in how CFIR constructs were used, and there was a trend of more studies investigating outcomes later in our review timeframe (2013–2014). Some studies used CFIR constructs as direct predictors of implementation effectiveness while others used CFIR constructs as control variables in investigating implementation outcomes. Unfortunately, a lack of specification of use of CFIR constructs did not allow us to synthesize findings across studies to see if there were trends in which CFIR constructs were found to influence outcomes, and under what conditions, revealing another gap in the research. Greater investigation of meaningful implementation outcomes, clearer linkage of CFIR constructs with outcomes, and clearer specification of which CFIR constructs were used to investigate outcomes would allow for more robust comparisons across studies. This would contribute to the field of implementation science by enabling structured investigation of which constructs influence outcomes and under what conditions.

### Contribution of the CFIR to implementation science

In the 6 years since the CFIR was published, its use in empirical studies has steadily increased and there is modest progress towards achieving the goals set forth by Damschroder et al. Terminology and language of the CFIR’s constructs appear to be coherent; the few suggestions for improvements to the CFIR did not point to significant changes in the framework. There was much less evidence of progress for the remaining two goals—promoting comparison of results across contexts and studies over time and advancing theory—but limited progress may be more a function of the short amount of time since the initial publication of the CFIR. In the 6 years since its publication, few studies have used the CFIR in a meaningful way (*n* = 26); however, this is understandable, given the number of years needed to conceive, obtain funding for, execute, and then publish research studies and their findings. Damschroder et al.’s goals were voiced earlier by developers of the PARIHS framework [27]. A review of application of the PARIHS framework approximately 10 years after its initial publication identified 18 articles describing empirical studies [28]. This review identified minimal prospective use of PARIHS in published articles and insufficient descriptions of how the framework was used to guide studies. The limitations identified in this review of the CFIR and of PARIHS are not unusual and reflect a general lack of integration of theory/frameworks into empirical studies [29, 30]. Better integration of the CFIR (or other theoretical framework) into empirical studies would help to address gaps in use of theory and advance implementation science [4, 31, 32]. Additionally, structured guidance on how to comprehensively apply theory in implementation research may help achieve consistency and rigor in the application of theory or frameworks when they are used by implementation researchers.

To assist researchers in their application of the CFIR, we have developed a list of recommendations (see Table 4). These include (1) consider how to most meaningfully use the CFIR across different phases of implementation, (2) report how CFIR constructs were selected and which constructs were used, (3) assess the association of CFIR constructs with outcomes, and (4) integrate the CFIR throughout the entire research process (e.g., to develop research questions, data collection materials, and to refine CFIR constructs and promote theoretical development). An online CFIR technical assistance website (www.cfirguide.org) is available to guide application of the CFIR in implementations and evaluations.

**Table 4:** Recommendations for applying the CIFR in implementation research

Recommendation	Rationale	Notes/explanation
Consider how to most meaningfully use the CFIR across different phases of implementation (pre-, during, or post-implementation).	Explicit use and reporting of CFIR constructs at various phases of implementation would allow comparisons across phases.	Meaningful pre-implementation assessment would help to identify barriers to address and facilitators to leverage, which in turn, would inform choice of strategies that will increase likelihood of implementation success. In addition, this information can be used to adapt the intervention to fit local context. Meaningful during or post-implementation studies would continue identification of barriers and facilitators to implementation to: ▪ Inform scale-up efforts ▪ Inform implementation of an innovation in other settings/contexts ▪ Associate CFIR constructs with implementation outcomes (if using mixed-methods or quantitative studies)
Report how CFIR constructs were selected for assessment.	Help to ensure rigor of implementation studies (i.e., that the most salient implementation-related factors were investigated) and promotes the ability to compare research over time and across contexts.	Researchers using the CFIR should clearly report: (1) justification/rationale for selecting CFIR constructs and (2) the CFIR constructs used in the research study.
Increase use of CFIR to investigate outcomes.	Investigation of outcomes allows for more robust comparisons across studies to identify which constructs influence outcomes and under what conditions.	Researchers should include measurement of implementation outcomes to assess association of CFIR constructs with implementation outcomes. Within the context of investigating outcomes, researchers should: (1) include implementation outcome measures (such as those mentioned by Proctor et al. [25]), (2) provide clearer linkage of CFIR constructs with outcomes, and (3) provide clearer specification of which CFIR constructs were used to investigate outcomes.
Integrate the CFIR more holistically into the research process.	Integrating the CFIR into research question development and data collection efforts early-on will strengthen research and applicability of findings.	The CFIR should be used throughout the research process. Researchers can use the CFIR Technical Assistance wiki (www.cfirguide.org) to develop research questions, interview and coding guides, as well as to refine CFIR constructs, definitions, and theoretical developments.

## Conclusions

In the 6 years since its publication, 26 of 429 published articles were identified as having used the CFIR in a meaningful way in a study. These articles collectively indicate that the CFIR has been used across a wide range of studies with a wide range of objectives, units of analysis, design, methods, and implementations of an array of innovations in an array of settings. However, more in-depth and prospective use of the CFIR (or other framework) may help to advance implementation science.

## Additional files

Additional file 1: Description of the five CFIR domains and constructs within each domain. (DOCX 25.0 kb)

Additional file 2: CFIR constructs used in studies, by author. (DOCX 14.5 kb)

## Abbreviations

CFIR: Consolidated Framework for Implementation Research
PARIHS: Promoting Action on Research Implementation in Health Services
