# Supplementary material for: A systematic review of the use of the Consolidated Framework for Implementation Research
Source: Implement Sci. 2016 May 17;11:72. doi: 10.1186/s13012-016-0437-z (PMC4869309; doi:10.1186/s13012-016-0437-z)
Supplement: Supplementary file 2 — CFIR constructs used in studies, by author. (DOCX 14.5 kb) [file 13012_2016_437_MOESM2_ESM.docx]

**CFIR constructs used in studies, by author**

| Domain | Constructs | Balas et al. (2013) [35] | Cilenti et al. (2012) [11] | Connell et al. (2014), *Phys Ther* [18] | Connell et al. (2014) *Impl Sci [36]* | Cragun et al. (2014) [19] | Damschroder and Lowery (2013) [20] | English et al. (2011) [38] | English et al. (2013) [22] | Forman et al. (2014) [17] | Gilmer et al. (2013) [39] | Ilott et al. (2012) [12] | Kilbourne et al. (2013) [21] | Sanchez et al. (2014) [41] | Shaw et al. (2013) [42] | Zulman et al. (2013) [43] | Count |
| --- | --- | --- | --- | --- | --- | --- | --- | --- | --- | --- | --- | --- | --- | --- | --- | --- | --- |
| Intervention |  |  |  |  |  |  |  |  |  |  |  |  |  |  |  |  |  |
|  | Intervention Source |  |  |  |  |  | X | X |  |  |  | X | X |  |  |  | 4 |
|  | Evidence Strength & Quality | X |  | X | X |  | X | X |  |  |  | X |  |  |  | X | 7 |
|  | Relative Advantage | X |  | X | X | X | X | X |  |  |  | X |  | X |  | X | 9 |
|  | Adaptability | X |  | X |  |  | X | X |  |  |  | X | X |  |  |  | 6 |
|  | Trialability |  |  |  |  | X | X | X |  |  |  | X |  | X |  | X | 6 |
|  | Complexity | X |  | X | X | X | X | X |  | X |  | X | X |  |  | X | 10 |
|  | Design Quality and Packaging |  |  |  | X |  | X | X |  |  |  | X |  |  |  | X | 5 |
|  | Cost |  |  |  |  | X | X | X |  |  |  | X |  |  |  | X | 5 |
| Outer Setting |  |  | X |  |  |  |  |  |  |  |  |  |  |  |  |  | 1 |
|  | Patient Needs/Resources | X |  |  |  | X | X | X |  |  | X | X |  |  |  | X | 7 |
|  | Cosmopolitanism |  |  |  | X |  | X | X | X |  | X | X |  |  |  |  | 6 |
|  | Peer Pressure |  |  |  |  | X | X | X | X |  |  | X | X |  |  | X | 7 |
|  | External Policy/Incentive |  |  |  |  | X | X | X |  |  | X | X |  | X |  | X | 7 |
| Inner Setting |  |  | X |  |  |  |  |  |  |  |  |  |  |  |  |  | 1 |
|  | Structural Characteristics |  |  |  |  |  | X | X |  |  |  | X | X |  |  | X | 5 |
|  | Networks & Communications | X |  |  |  | X | X | X | X | X | X | X |  | X |  |  | 9 |
|  | Culture | X |  |  |  |  | X | X |  |  | X | X | X |  |  | X | 7 |
|  | Implementation Climate: Tension for Change |  |  |  |  |  | X | X |  |  |  | X |  |  |  |  | 3 |
|  | Implementation Climate: Compatibility |  |  |  |  |  | X | X |  | X | X | X | X | X |  |  | 7 |
|  | Implementation Climate: Relative Priority |  |  |  |  |  | X | X |  |  |  | X | X |  |  |  | 4 |
|  | Implementation Climate: Org Incentives/Rewards |  |  |  |  |  | X | X |  |  |  | X |  |  |  |  | 3 |
|  | Implementation Climate: Goals/Feedback |  |  |  |  |  | X | X | X |  |  | X |  |  |  |  | 4 |
|  | Implementation Climate: Learning Climate |  |  |  |  |  | X | X | X |  |  | X |  | X |  |  | 5 |
|  | Readiness for Implementation: Leadership engagement |  |  |  | X |  | X | X | X |  |  | X |  |  |  |  | 5 |
|  | Readiness for Implementation: Available Resources |  | X |  |  |  | X | X |  | X |  | X | X | X |  |  | 7 |
|  | Readiness for Implementation: Access to Knowledge/Information |  |  |  | X | X | X | X |  | X |  | X |  |  |  |  | 6 |
| Characteristics of Individuals |  |  |  |  |  |  |  |  |  |  |  |  |  |  |  |  |  |
|  | Knowledge and Beliefs about Intervention |  |  | X | X | X | X | X |  |  | X | X | X | X | X | X | 11 |
|  | Self-efficacy | X |  | X |  |  | X | X | X |  |  | X | X | X | X | X | 10 |
|  | Individual Stage of change |  |  |  |  |  | X | X |  |  |  | X |  | X |  |  | 4 |
|  | Individual Identification with Organization |  |  | X |  |  | X | X |  |  |  | X |  |  |  | X | 5 |
|  | Other Personal Attributes |  |  | X |  |  | X | X |  |  | X | X |  | X |  |  | 6 |
| Process |  |  | X |  |  |  |  |  |  |  |  |  |  |  |  |  | 1 |
|  | Planning | X |  |  |  | X | X | X |  |  |  | X |  | X |  | X | 7 |
|  | Engaging: Opinion Leaders |  |  |  |  |  | X | X |  |  |  | X |  | X |  |  | 4 |
|  | Engaging: Formally appointed internal implementation leaders |  |  |  |  |  | X | X |  |  |  | X |  |  |  |  | 3 |
|  | Engaging: champions |  |  |  |  |  | X | X |  |  |  | X |  | X |  |  | 4 |
|  | Engaging: external Change agents |  |  |  |  |  | X | X |  |  |  | X |  |  |  |  | 3 |
|  | Executing | X |  |  |  |  | X | X |  |  |  | X | X | X |  | X | 7 |
|  | Reflecting & Evaluating | X |  |  |  |  | X | X |  |  |  | X |  | X |  | X | 6 |

Note: Only 15 of the 26 included articles are reflected in this table because this table reflects only articles which explicitly specified CFIR constructs used in their research. Thus, 9 articles were excluded from this table (Acosta et al. [33], Baker et al. [34], Draanen et al. [37], Green et al. [23], Jones et al. [40], Kalkan et al. [9], Luck et al. [16], Robins et al. [10], and Ruffolo & Capobianco [13]) because each only referenced or used CFIR domains and no constructs. Two articles (Shimada et al. [14] and Zulman et al. [15]) were excluded from this table because neither study made explicit reference to any CFIR constructs or domains.
